# No improvement in clinical outcomes: a propensity score analysis of inappropriate carbapenem use in children with susceptible *Escherichia coli* bloodstream infections

**DOI:** 10.3389/fcimb.2026.1829667

**Published:** 2026-06-02

**Authors:** Qian Zhang, Ya Liu, Junming Huo, Kaibin Pu, Liping Tan, Jie Cheng

**Affiliations:** 1 Department of Pediatrics, Chongqing Youyoubaobei Women and Children’s Hospital, Chongqing, China; 2 Department of Intensive Care Unit, Children’s Hospital of Chongqing Medical University, National Clinical Research Center for Children and Adolescents’ Health and Diseases, Ministry of Education Key Laboratory of Child Development and Disorders, Intelligent Application of Big Data in Pediatrics Engineering Research Center of Chongqing Education Commission of China, Chongqing, China; 3 Chongqing Key Laboratory of Child Rare Diseases in Infection and Immunity, Chongqing, China; 4 Department of Emergency, Children’s Hospital of Chongqing Medical University, National Clinical Research Center for Children and Adolescents’ Health and Diseases, Ministry of Education Key Laboratory of Child Development and Disorders, Intelligent Application of Big Data in Pediatrics Engineering Research Center of Chongqing Education Commission of China, Chongqing, China

**Keywords:** clinical outcomes, *Escherichia coli* bloodstream infection, inappropriate carbapenem use, pediatric infection, propensity score matching

## Abstract

**Objective:**

To determine whether carbapenem overuse affects clinical outcomes in children with susceptible *Escherichia coli* (*E. coli*) bloodstream infection (BSI) using propensity score matching (PSM).

**Methods:**

We conducted a retrospective study of 206 pediatric patients with *E. coli* BSI. This study evaluated the appropriateness of empirical antibiotic therapy (initiated before blood culture results), retrospectively judged by subsequent susceptibility testing. Patients were categorized into a standard-therapy group (n=123) and an inappropriate carbapenem therapy group (n=83). One-to-one PSM generated 67 matched pairs with balanced baseline characteristics. Standard logistic regression was adopted for correlation analyses before matching, while conditional logistic regression was used to explore risk associations with mortality, sepsis and septic shock after matching.

**Results:**

After PSM, all covariates were balanced (standardized mean differences <0.2). Overall mortality was 10.68%. No significant differences were observed between the inappropriate carbapenem group and the standard therapy group regarding mortality, sepsis, septic shock, or duration of hospitalization, in both unmatched and matched analyses. In the matched cohort, no significant associations were observed for mortality (OR 0.75, 95% CI 0.26-2.16, p= 0.594), sepsis (OR 0.93, 95% CI 0.44-1.98, p=0.847), septic shock (OR 0.83, 95% CI 0.25-2.73, p=0.776). Likewise, no significant between-group difference in duration of hospitalization was detected via the paired Wilcoxon signed-rank test (p = 0.777).

**Conclusions:**

No significant differences in mortality, sepsis, septic shock, or duration of hospitalization were observed between the inappropriate carbapenem group and the standard therapy group. These findings reinforce the importance of antimicrobial stewardship by demonstrating that initial narrow-spectrum therapy (third-generation cephalosporins) achieves comparable outcomes, thereby reducing unnecessary carbapenem exposure.

## Introduction

Antimicrobial resistance among gram-negative pathogens has emerged as a major global health threat (Furlan et al., 2025). Carbapenem-resistant Enterobacterales, in particular, are associated with high mortality, limited therapeutic options, and substantial healthcare burden (Furlan et al., 2025). In response to rising resistance rates, carbapenems have increasingly been used as empirical therapy for severe infections, including bloodstream infections (BSIs) caused by *Escherichia coli* (*E. coli*) (Walker et al., 2024). However, carbapenems are still widely used even in non-critical patients (Walker et al., 2024).

Although carbapenems remain highly effective against extended-spectrum beta-lactamases (ESBLs)-producing organisms, their widespread use has contributed to escalating selective pressure and the global dissemination of carbapenem-resistant strains (Patrier and Timsit, 2020). In many settings, carbapenem exposure itself is a key driver of resistance development (Patrier and Timsit, 2020). Consequently, optimizing carbapenem use has become a critical component of international strategies to combat antimicrobial resistance.

In pediatric populations, *E. coli* is one of the most common causes of BSI (Belmont-Monroy et al., 2025). Children with malignancies, immunosuppression, or prolonged hospitalization are at particular risk. Due to concerns about resistant pathogens and clinical instability, carbapenems are frequently administered empirically (Mihalcea et al., 2023; Castagnola et al., 2024) and sometimes continued even after susceptibility testing demonstrates that narrower-spectrum antibiotics are adequate (Poline et al., 2021). However, it remains unclear whether such carbapenem overuse improves clinical outcomes in infections caused by susceptible isolates.

Clarifying this question is essential in the context of the global resistance crisis. If inappropriate carbapenem use does not improve prognosis, unnecessary exposure may represent avoidable selective pressure without clinical benefit. Observational evaluations of antibiotic appropriateness are subject to confounding, particularly confounding by severity. Propensity score matching (PSM) offers a robust statistical approach to balance baseline differences and approximate causal inference.

In this study, we examined the impact of inappropriate carbapenem therapy on mortality, sepsis, septic shock, and length of hospital stay in children with carbapenem-susceptible *E. coli* BSI. Using a propensity score-matched design, we aimed to determine whether carbapenem overuse provides measurable clinical advantage or instead represents potentially avoidable antimicrobial exposure in pediatric care.

## Methods

### Study designs and patients

This retrospective cohort study was granted a waiver of informed consent by the ethics committee. Conducted at the Children’s Hospital of Chongqing Medical University, a National Clinical Research Center in China, the study enrolled pediatric inpatients diagnosed with *E. coli* BSI between 2015 and 2021. The inclusion criteria were: (i) non-neonatal inpatients; and (ii) confirmation of *E. coli* in blood culture. Exclusion criteria comprised: (i) with incomplete essential laboratory data; (ii) with incomplete clinical information; (iii) presence of polymicrobial BSI; (iv) received antibiotics other than third-generation cephalosporins or carbapenems. As this study did not involve any human or animal experiments, formal approval was obtained from the Ethics Committee of the Children’s Hospital of Chongqing Medical University (Approval No. 2023-423). All procedures were performed in accordance with the principles of the Declaration of Helsinki.

### Data collection and definitions

Data collection encompassed demographic characteristics, laboratory findings, imaging results, underlying conditions, diagnoses, treatment modalities, complications, and clinical outcomes. C-reactive protein (CRP) was measured from a blood sample drawn simultaneously with the first blood culture that later turned positive. Time to positivity (TTP) was recorded from the same culture specimen after incubation. However, some patients had received antibiotics prior to blood culture collection, which could potentially affect both CRP levels and TTP. To avoid overmatching bias and to address concerns about intermediate variables, we performed sensitivity analyses excluding TTP from the propensity score model; the results were unchanged (**Supplementary Tables 1, 2**). All baseline covariates, including prior antibiotic exposure, were balanced after PSM.

BSI was defined as the presence of positive blood cultures accompanied by systemic signs of infection (Cheng et al., 2020). Hospital-acquired infection was defined as infection occurring more than 48 hours after hospital admission (Cheng et al., 2020). According to a published study from our region (Chongqing, China) (Gu and Jing, 2017), the average ESBL detection rate among *E. coli* isolates from children in Chongqing was 50.98% during 2010-2015, partially overlapping with our study period. Inappropriate carbapenem use was defined as empirical administration of carbapenems initiated before blood culture results were available in patients whose isolates were subsequently shown to be susceptible to third-generation cephalosporins (Deelen et al., 2021). Susceptibility was determined based on Clinical and Laboratory Standards Institute (CLSI) breakpoints (Clinical and Laboratory Standards Institute, 2014), and isolates with evidence of ESBLs production were considered resistant (Mark et al., 2021). Chemotherapy-induced bone marrow suppression was diagnosed following World Health Organization (WHO) criteria (Miller et al., 1981). TTP was defined as the interval between blood specimen incubation and the positive signal alert from the automated blood culture system (Cheng et al., 2020). Sepsis and septic shock were diagnosed according to the criteria outlined in the Surviving Sepsis Campaign: International Guidelines for Management of Sepsis and Septic Shock 2021 (Evans et al., 2021).

### Exposure and outcomes

The primary exposure of interest was defined as the inappropriate use of carbapenems following blood culture collection. The primary outcome was all-cause in-hospital mortality. The secondary outcomes included the occurrence of sepsis and septic shock, as well as the total duration of hospitalization.

### Propensity score matching analysis

PSM was used to balance baseline covariates and reduce model dependence. To assess baseline covariate balance, standardized mean differences (SMDs) and hypothesis-testing p-values were calculated for the primary variables. Propensity scores were estimated using logistic regression based on the following baseline covariates: age, sex, TTP, CRP, bone marrow suppression after chemotherapy, malignancy, hospital-acquired infection, prior antimicrobial administration before blood culture, and invasive mechanical ventilation. One-to-one nearest-neighbor matching without replacement was performed with a caliper width of 0.2 times the standard deviation of the logit of the propensity score. Covariate balance before and after matching was assessed using SMDs, with an SMD <0.1 indicating adequate balance and SMDs between 0.1 and 0.2 representing small differences unlikely to require further adjustment, as suggested by Austin (2011). Balance diagnostics were further visualized using Love plots. After matching, the robustness of the effect estimates to unmeasured confounding was evaluated through Rosenbaum sensitivity analysis, E-value estimation, and simulation of hypothetical unmeasured confounders across a range of confounding strengths (odds ratio [OR] from 1.0 to 3.0), covering scenarios from no confounding to strong confounding that could potentially alter the study conclusions, as commonly explored in sensitivity analyses (Groenwold et al., 2010).

### Statistical analysis

Continuous variables were summarized as median (interquartile range, IQR), and categorical variables were expressed as counts and percentages. Between-group comparisons for continuous variables were performed using the Mann-Whitney U test (also known as the Wilcoxon rank-sum test), while categorical variables were compared using the χ² test or Fisher’s exact test, as appropriate. After one-to-one propensity score matching, paired Wilcoxon signed-rank test was adopted for continuous variable comparison. Binary clinical outcomes were assessed via conditional logistic (clogit) regression, and the results were presented as odds ratios (ORs) and 95% confidence intervals (CIs). In addition, we performed visualized analyses to intuitively exhibit the study results. Box plots were generated to display the distribution of hospitalization duration before and after PSM, and forest plots were constructed to summarize the effect sizes of the associations between inappropriate carbapenem use and clinical outcomes. All statistical analyses were performed using R software (version 4.3.0; R Foundation for Statistical Computing). A two-sided *P* value <0.05 was considered statistically significant.

## Results

### Study population

**Figure 1:**
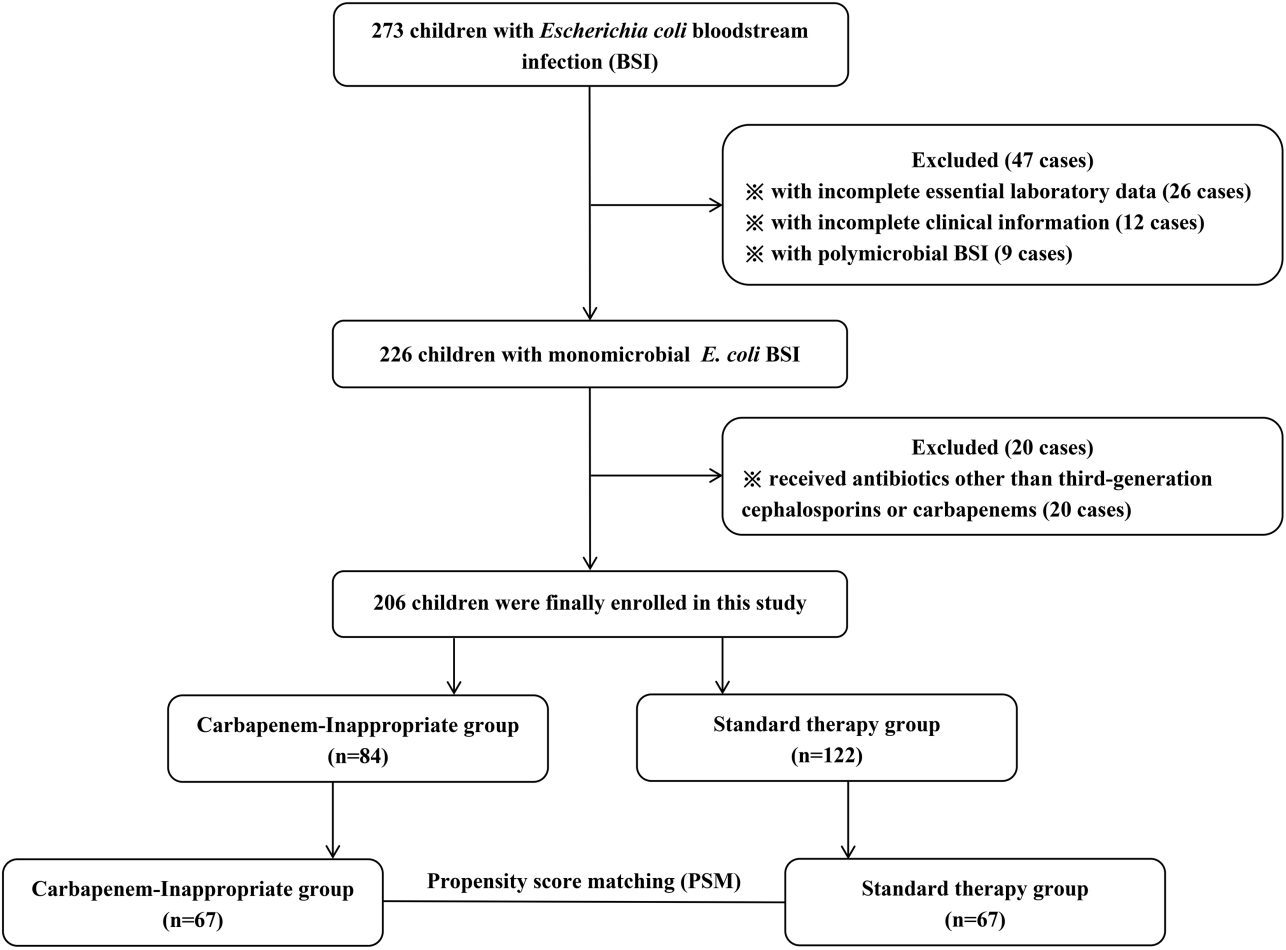
Flowchart of this study.

Initially, 273 patients were considered. After excluding 67 patients due to incomplete essential laboratory data (n=26), use of antibiotics other than third-generation cephalosporins or carbapenems (n=20), incomplete clinical information (n=12), or polymicrobial bloodstream infection (n=9), a total of 206 patients were enrolled in the study. These patients were subsequently divided into two groups based on the initial treatment received: the standard-therapy group (n=123) and the carbapenem-inappropriate group (n=83), and their clinical characteristics were compared. The patient selection process was illustrated in **Figure 1**.

### Characteristics of 206 children with *Escherichia coli* BSI

A total of 206 children with *E. coli* BSI were included. Their demographic, laboratory, and clinical characteristics were summarized in **Table 1**. Briefly, the median age was 1.84 (IQR 0.22–8.83) years, 60.68% were male. Regarding clinical outcomes, overall mortality was 10.68%, sepsis occurred in 26.21%, septic shock in 8.25%, and the median duration of hospitalization was 23.92 (IQR, 13.48-35.71) days.

**Table 1:** Characteristics of 206 children with *E. coli* bloodstream infection (BSI).

Characteristics	Total (n=206)
Demographic data	
Age (years) (median, IQR)	1.84 (0.22-8.83)
Sex (male) (n, %)	125 (60.68%)
Patterns of admission	
Admitted through the Emergency Department (n, %)	27 (13.11%)
Admitted via transfer from another hospital (n, %)	29 (14.08%)
Laboratory data	
C-reactive protein (CRP) (mg/L) (median, IQR)	37.00 (15.25-65.75)
Procalcitonin (PCT) (ng/ml) (median, IQR)	2.15 (0.31-17.89)
Albumin level (g/L) (median, IQR)	35.70 (30.83-40.70)
Underlying condition	
Bone marrow inhibition after chemotherapy (n, %)	104 (50.49%)
Malignancy (n, %)	99 (48.06%)
Congenital heart disease (n, %)	29 (14.08%)
Surgery history in one year (n, %)	16 (7.77%)
Malnutrition (n, %)	4 (1.94%)
Antimicrobial agents administrated before blood culture (n, %)	58 (28.16%)
Susceptible antimicrobial agents administrated before blood culture (n, %)	20 (9.71%)
Antimicrobial therapy after blood culture	
Carbapenems (n, %)	160 (77.67%)
Third-generation cephalosporins (n, %)	46 (22.33%)
Infection of unknown origin (n, %)	108 (52.43%)
Hospital-acquired infection (n, %)	93 (45.15%)
Inappropriate use of carbapenems (n, %)	83 (40.29%)
Invasive mechanical ventilation (n, %)	18 (8.74%)
Pediatric sequential organ failure assessment (pSOFA) score (median, IQR)	1.00 (0.00-2.00)
Time to positivity (TTP) (hours) (median, IQR)	14.93 (12.88-17.88)
Primary outcomes	
Mortality (n, %)	22 (10.68%)
Secondary outcomes	
Sepsis (n, %)	54 (26.21%)
Septic shock (n, %)	17 (8.25%)
Duration of hospitalization (days) (median, IQR)	23.92 (13.48-35.71)

### Comparisons of clinical characteristics between standard therapy and inappropriate carbapenem therapy groups before and after PSM

Before PSM, the inappropriate carbapenem group had significantly older age, higher rates of bone marrow suppression, malignancy, and hospital-acquired infection (see **Table 2** for detailed comparisons). After 1:1 PSM, 67 matched pairs were generated, and all baseline characteristics were well balanced between the two groups (all SMDs < 0.2; **Table 3**; **Supplementary Figures 1, 2**).

**Table 2:** Comparison of characteristics between the standard therapy group and the inappropriate carbapenem therapy group in 206 children with *E. coli* BSI before propensity score matching (PSM).

Characteristics	Standard-therapy group (n=123)	Carbapenem-inappropriate group (n=83)	P-value	SMD
Demographic data				
Age (years) (median, IQR)	0.79 (0.19-5.38)	5.67 (0.51-11.84)	<0.001*	0.625
Sex (male) (n, %)	78 (63.41%)	47 (56.63%)	0.405	0.139
Patterns of admission				
Admitted through the Emergency Department (n, %)	13 (10.57%)	14 (16.87%)	0.270	0.184
Admitted via transfer from another hospital (n, %)	19 (15.45%)	10 (12.05%)	0.629	0.099
Laboratory data				
CRP (mg/L) (median, IQR)	47.00 (16.50-79.00)	33.00 (15.00-50.50)	0.061	0.366
PCT (ng/ml) (median, IQR)	2.39 (0.31-18.21)	2.01 (0.33-16.95)	0.557	0.177
Albumin level (g/L) (median, IQR)	35.80 (31.05-40.60)	35.70 (30.25-41.20)	0.678	0.026
Underlying condition				
Bone marrow inhibition after chemotherapy (n, %)	50 (40.65%)	54 (65.06%)	0.001*	0.504
Malignancy (n, %)	48 (39.02%)	51 (61.45%)	0.003*	0.460
Congenital heart disease (n, %)	17 (13.82%)	12 (14.46%)	1.000	0.018
Surgery history in one year (n, %)	12 (9.76%)	4 (4.82%)	0.302	0.191
Malnutrition (n, %)	2 (1.63%)	2 (2.41%)	1.000	0.056
Antimicrobial agents administrated before blood culture (n, %)	41 (33.33%)	17 (20.48%)	0.064	0.293
Susceptible antimicrobial agents administrated before blood culture (n, %)	12 (9.76%)	8 (9.64%)	1.000	0.004
Infection of unknown origin (n, %)	60 (48.78%)	48 (57.83%)	0.257	0.182
Hospital-acquired infection (n, %)	46 (37.40%)	47 (56.63%)	0.010*	0.393
Invasive mechanical ventilation (n, %)	11 (8.94%)	7 (8.43%)	1.000	0.018
pSOFA score (median, IQR)	1.00 (0.00-2.00)	1.00 (0.00-1.50)	0.756	0.014
TTP (hours) (median, IQR)	15.12 (13.42-18.37)	14.78 (12.71-17.17)	0.126	0.302

*with statistical significance, P <0.05.

**Table 3:** Comparison of characteristics between the standard therapy group and inappropriate carbapenem therapy group in children with *E. coli* BSI after PSM.

Characteristics	Standard-therapy group (n=67)	Carbapenem-inappropriate group (n=67)	P-value	SMD
Demographic data				
Age (years) (median, IQR)	2.67 (0.26-9.41)	3.50 (0.23-9.16)	0.878	0.058
Sex (male) (n, %)	36 (53.73%)	37 (55.22%)	1.000	0.030
Patterns of admission				
Admitted through the Emergency Department (n, %)	9 (13.43%)	12 (17.91%)	0.635	0.123
Admitted via transfer from another hospital (n, %)	6 (8.96%)	9 (13.43%)	0.584	0.142
Laboratory data				
CRP (mg/L) (median, IQR)	35.00 (13.00-62.50)	37.00 (15.50-52.50)	0.957	0.084
PCT (ng/ml) (median, IQR)	1.31 (0.21-10.88)	2.09 (0.52-17.77)	0.109	0.128
Albumin level (g/L) (median, IQR)	36.90 (30.90-41.60)	35.10 (30.25-39.80)	0.461	0.088
Underlying condition				
Bone marrow inhibition after chemotherapy (n, %)	38 (56.72%)	39 (58.21%)	1.000	0.030
Malignancy (n, %)	37 (55.22%)	37 (55.22%)	1.000	<0.001
Congenital heart disease (n, %)	7 (10.45%)	9 (13.43%)	0.790	0.092
Surgery history in one year (n, %)	6 (8.96%)	4 (5.97%)	1.000	0.060
Malnutrition (n, %)	2 (2.99%)	2 (2.99%)	1.000	0.101
Antimicrobial agents administrated before blood culture (n, %)	17 (25.37%)	15 (22.39%)	0.839	0.070
Susceptible antimicrobial agents administrated before blood culture (n, %)	4 (5.97%)	6 (8.96%)	0.742	0.114
Infection of unknown origin (n, %)	41 (61.19%)	38 (56.72%)	0.725	0.091
Hospital-acquired infection (n, %)	35 (52.24%)	36 (53.73%)	1.000	0.030
Invasive mechanical ventilation (n, %)	6 (8.96%)	5 (7.46%)	1.000	0.054
pSOFA score (median, IQR)	1.00 (1.00-1.50)	1.00 (0.00-1.50)	0.825	0.026
TTP (hours) (median, IQR)	14.93 (13.39-17.26)	14.65 (12.56-17.03)	0.424	0.024

*with statistical significance, P <0.05.

### Clinical outcomes before and after PSM in children with *E. coli* BSI

No significant differences in primary or secondary outcomes were observed between the standard-therapy group and the inappropriate carbapenem group before or after PSM (all p > 0.05). Detailed outcome data, including mortality rates, sepsis, septic shock, and duration of hospitalization, were presented in **Table 4**.

**Table 4:** Clinical outcomes of children with *E. coli* BSI before and after propensity score matching (PSM).

Outcomes	Before PSM			After PSM		
	Standard-therapy group (n=123)	Carbapenem-inappropriate group (n=83)	P-value	Standard-therapy group (n=67)	Carbapenem-inappropriate group (n=67)	P-value
Primary outcome						
Mortality (n, %)	13 (10.57%)	9 (10.84%)	1.000	9 (13.43%)	7 (10.45%)	0.790
Secondary outcomes						
Sepsis (n, %)	34 (27.64%)	20 (24.10%)	0.685	17 (25.37%)	16 (23.88%)	1.000
Septic shock (n, %)	9 (7.32%)	8 (9.64%)	0.737	6 (8.96%)	5 (7.46%)	1.000
Duration of hospitalization (days) (median, IQR)	18.92 (12.59-35.75)	26.92 (15.40-35.44)	0.067	26.96 (12.59-39.00)	23.92 (15.39-34.94)	0.776

### Sensitivity analyses and post-matching balance assessment

After PSM, the mean absolute SMD was 0.045 (**Supplementary Figure 3C**). Rosenbaum bounds analysis showed that an unmeasured confounder would need to increase the odds of exposure by more than 2.1-fold to potentially alter the conclusion (**Supplementary Figure 3A**). Simulation of hypothetical unmeasured confounders across a range of strengths yielded consistently non-significant adjusted ORs (**Supplementary Figure 3B**).

### Association between inappropriate carbapenem use and clinical outcomes pre- and post-PSM

**Figure 2:**
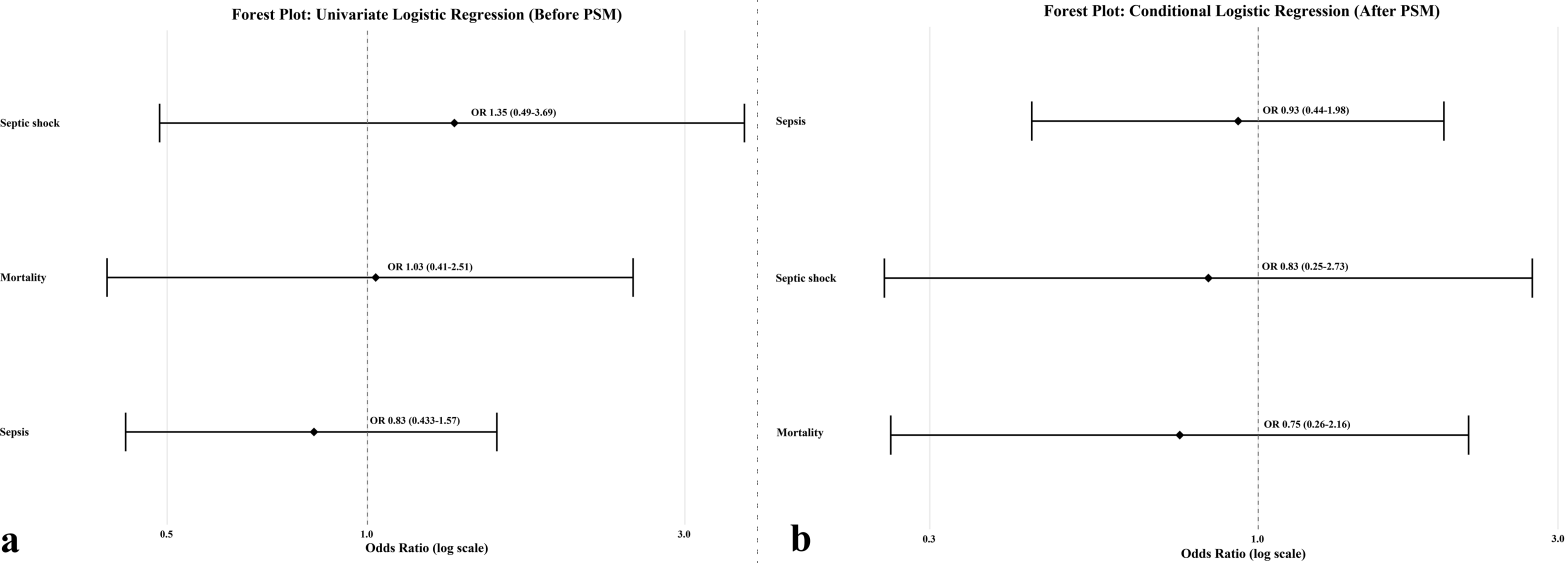
Association between inappropriate carbapenem use and clinical outcomes before and after propensity score matching (PSM). **(a)** Univariate analysis before PSM. **(b)** Univariate analysis after PSM.

**Figure 3:**
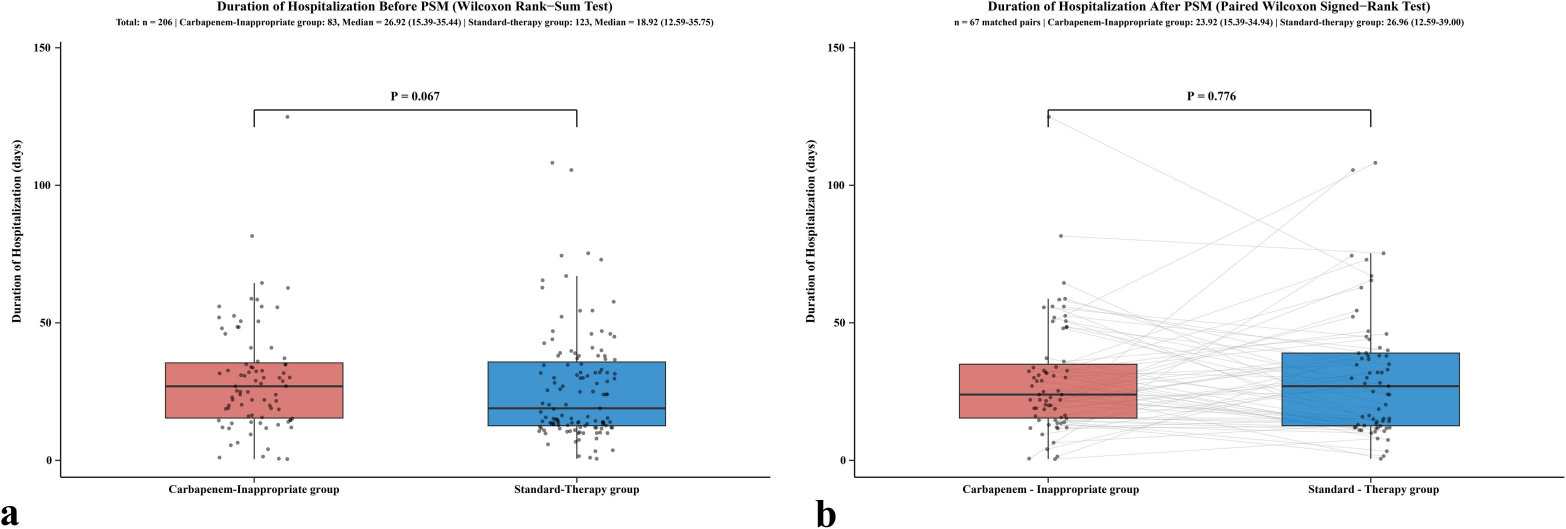
Distribution of duration of hospitalization in children with *E. coli* BSI before and after 1:1 PSM. **(a)** Before PSM, compared using the Wilcoxon rank-sum test. **(b)** After PSM, compared using the paired Wilcoxon signed-rank test, with gray lines representing matched pairs.

In pre-matching analysis, inappropriate carbapenem use was not significantly associated with mortality (OR 1.03, 95% CI 0.41-2.51, p=0.950), sepsis (OR 0.83, 95% CI 0.43-1.57, p=0.571), or septic shock (OR 1.35, 95% CI 0.49-3.69, p=0.554). For duration of hospitalization, the Wilcoxon rank-sum test showed no significant difference between the two groups (p = 0.067). Details were shown in **Table 5**; **Figures 2a**, **3a**.

**Table 5:** Association between inappropriate carbapenem use and clinical outcomes before and after PSM.

PSM	Outcomes	Model	Effect	P-value
Pre-matching	Mortality	Logistic	OR 1.03 (0.41-2.51)	0.950
Post-matching	Mortality	Conditional logistic regression (clogit)	OR 0.75 (0.26-2.16)	0.594
Pre-matching	Sepsis	Logistic	OR 0.83 (0.43-1.57)	0.571
Post-matching	Sepsis	clogit	OR 0.93 (0.44-1.98)	0.847
Pre-matching	Septic shock	Logistic	OR 1.35 (0.49-3.69)	0.554
Post-matching	Septic shock	clogit	OR 0.83 (0.25-2.73)	0.776

After propensity score matching, inappropriate carbapenem use remained not significantly associated with mortality (OR 0.75, 95% CI 0.26-2.16, p= 0.594), sepsis (OR 0.93, 95% CI 0.44-1.98, p=0.847), septic shock (OR 0.83, 95% CI 0.25-2.73, p=0.776). For duration of hospitalization, the paired Wilcoxon signed-rank test showed no significant difference between the two groups (p = 0.776). Details were presented in **Table 5**; **Figures 2b**, **3a**.

## Discussion

This study used PSM to systematically analyze the association between inappropriate carbapenem use and clinical outcomes of *E. coli* BSI in children. It compared the standard therapy group (which received third-generation cephalosporins as either initial or definitive therapy, without inappropriate carbapenem continuation) and the inappropriate carbapenem use group (which received carbapenems despite susceptibility to cephalosporins). The key finding is that no statistically significant differences were observed between the two groups in mortality, sepsis, septic shock, or duration of hospitalization, either before matching or after 1:1 PSM. In other words, treating children with susceptible *E. coli* BSI with third-generation cephalosporins (i.e., starting with a narrower spectrum) was associated with similar short-term outcomes as starting with or continuing carbapenems.

This result directly supports antimicrobial stewardship programs that encourage initial use of narrower-spectrum agents when the pathogen is known or strongly suspected to be susceptible. It also aligns with the conclusions of Georges et al. (2022), but differs from the subjective perception among some clinicians that inappropriate carbapenem use necessarily leads to poor treatment outcomes (Zilberberg et al., 2017).

*E. coli* is one of the leading pathogens of community-acquired and hospital-acquired bloodstream infections in children (Belmont-Monroy et al., 2025). Due to the immature immune systems of children, coupled with high-risk factors such as underlying diseases and malnutrition in some patients, *E. coli* BSI can easily progress to sepsis, septic shock, and even death. Therefore, the rational selection of antimicrobial drugs is a crucial step in improving clinical outcomes for children. Carbapenems, as broad-spectrum and potent antimicrobial agents, possess strong antibacterial activity against Gram-negative bacteria, including *E. coli*, and are important drugs for the clinical treatment of moderate to severe Gram-negative bacterial infections. However, their inappropriate use has become increasingly prominent, not only because it may induce carbapenem resistance (Elshamy and Aboshanab, 2020), and disrupt the intestinal flora (Patangia et al., 2022), but also because it provides no clinical advantage over narrower-spectrum agents when the isolate is susceptible. Thus, clarifying whether initial carbapenem therapy offers any benefit over initial cephalosporin therapy in this setting is essential for pediatric infectious diseases.

Our finding that initial treatment with third-generation cephalosporins achieved comparable outcomes to carbapenems has several underlying explanations. First, this study rigorously corrected for baseline characteristics in both groups using the PSM method, effectively balancing the influence of potential confounding factors such as age, sex, underlying diseases, and infection severity. This eliminated differences in outcomes between groups due to baseline imbalances, more accurately reflecting the effect of carbapenem administration itself on clinical outcomes-a crucial statistical basis for our results. Second, the clinical outcome of *E. coli* BSI in children is the result of multiple factors, including pathogen characteristics, host immune status, anti-infective therapy, and supportive care, and is not determined by the choice of a single antibiotic (Bergin et al., 2015). In this study, the standardized treatment group used antibiotics selected based on clinical guidelines and initial drug sensitivity results of the pathogens. Although not carbapenems, these antibiotics were still effective against *Escherichia coli*, achieving effective control of the infection. In contrast, the inappropriate use of carbapenems in the group primarily involved overly broad indications, such as using carbapenems for mild infections or for infections that could have been treated with narrower-spectrum agents. This was not due to insufficient dosage, inappropriate treatment duration, or selection of resistant drugs. Therefore, no treatment failure occurred due to inappropriate drug use, which is the core clinical reason for the lack of significant difference in clinical outcomes between the two groups. Furthermore, all children in this study received standardized comprehensive treatment, including fluid resuscitation, administration of vasoactive drugs, nutritional support, and symptomatic treatment. This comprehensive supportive care effectively improved the children’s overall condition, reduced the risk of serious complications, and to some extent offset the potential negative impacts of inappropriate antibiotic selection, ensuring overall treatment efficacy (Paul et al., 2023).

Crucially, our results should not be interpreted as a justification for inappropriate carbapenem use. On the contrary, the absence of any clinical advantage over cephalosporins reinforces the message that starting with a narrower spectrum - i.e., using third-generation cephalosporins as initial therapy when susceptibility is likely - is safe and does not compromise short-term outcomes. Antimicrobial stewardship programs should continue to target unnecessary carbapenem exposure, not because it causes immediate harm, but because the long-term risks - selection of resistance and microbiota disruption (Elshamy and Aboshanab, 2020; Patangia et al., 2022) - far outweigh any perceived benefit. Our findings support the practice of beginning with narrower agents whenever clinically appropriate, rather than defaulting to carbapenems.

From a clinical practice perspective, this study provides important practical references for antimicrobial management in pediatric *E. coli* BSI. Clinicians may consider initiating third-generation cephalosporins in children with suspected or confirmed susceptible *E. coli* BSI, especially in settings with moderate ESBL prevalence. The focus could be on early pathogen identification, timely susceptibility testing, individualized anti-infective plans, management of underlying conditions, and early intervention for serious complications. The principle of “starting narrow” is recommended: narrow-spectrum, low-toxicity antibiotics could be used as initial therapy when the isolate is known or likely to be susceptible, reserving carbapenems for cases with documented resistance, moderate-to-severe infections where risk of resistance is high, or patients who fail initial narrow-spectrum therapy. This balance between effective treatment and antibiotic stewardship is critical.

Meanwhile, the results of this study echo some previous related research findings. Some studies on adult patients have also found no significant association between inappropriate use of carbapenems and short-term clinical outcomes (Muhammed et al., 2017; Yamaguchi et al., 2022; Yoo et al., 2023; Ngiam et al., 2025). The core reason for this is that inappropriate use often manifests as overly broad indications rather than therapeutic inappropriateness, and the effect of comprehensive treatment offsets the potential impact of drug selection. However, unlike adult studies, children, as a special population, exhibit significant differences in the pharmacokinetic characteristics (Tanaka et al., 2025) and immune status (Shekhar and Petersen, 2020) of antimicrobial drugs compared to adults. The results of this study fill a gap in related research in the pediatric population, providing evidence-based medicine for the individualized use of antimicrobial drugs in pediatrics. However, it should also be noted that different studies differ in population characteristics, treatment guidelines, and the definition of inappropriate carbapenem use.

The discrepancy in findings may be partly attributable to differences in study populations. Some previous studies that reported an association between inappropriate carbapenem use and adverse outcomes primarily enrolled patients with a high prevalence of multidrug-resistant infections (Rodríguez et al., 2024; Cornistein et al., 2025) or cases involving severely inappropriate use (Soneda et al., 2023; Wang et al., 2024). Therefore, the results of this study cannot be generalized, and clinicians still need to make individualized judgments based on specific case circumstances. Our findings are most directly applicable to settings with an ESBL prevalence similar to that in our region [approximately 50%, as reported by Gu et al (Gu and Jing, 2017)]. In settings with very low ESBL rates, empirical cephalosporins would be even safer; in settings with extremely high resistance rates, empirical carbapenems may still be warranted, and our results apply primarily to the de-escalation step after susceptibility is confirmed.

This study has several limitations. First, as a single-center retrospective study, it is subject to selection and information bias; the assessment of antibiotic appropriateness and the completeness of clinical data may have influenced the accuracy of the results. Second, the relatively small sample size and single-center design resulted in a degree of homogeneity in patient baseline characteristics and treatment regimens, limiting the generalizability of our findings. Third, we cannot rule out that undocumented concurrent infections at other sites, or clinician-perceived severity not captured by our covariates, may have influenced the decision to continue carbapenems. Such factors could represent unmeasured confounding and are a limitation of our retrospective design. Fourth, we focused only on short-term outcomes such as mortality and length of hospital stay, without evaluating the long-term impact of carbapenem exposure on children’s gut microbiota, subsequent antimicrobial resistance, or risk of reinfection. Fifth, although propensity score matching was applied to adjust for measurable confounders, residual confounding from unmeasured factors-such as specific combination therapy regimens and variations in supportive care intensity-cannot be ruled out. Sixth, while our exposure was empirical (before culture results), the safety of empirical narrow-spectrum therapy in high-risk children requires further study, as the standard-therapy group included patients who were lucky to have susceptible isolates. Moreover, even after PSM, residual differences in empirical starting risks (e.g., clinician-perceived severity at the time of empirical prescription) and subsequent treatment variations (e.g., timing of de-escalation among those initially started on carbapenems) cannot be fully excluded. To address these limitations, future multicenter prospective cohort studies with larger sample sizes are needed to enable subgroup analyses stratified by resistance phenotype, to incorporate long-term follow-up assessments, and to specifically collect data on all concurrent infections to better address this source of potential confounding. Furthermore, well-designed randomized controlled trials directly comparing carbapenems with other susceptible antibiotics would provide higher-quality evidence to guide antibiotic selection for pediatric *E. coli* BSIs and promote rational antimicrobial use in this vulnerable population.

Although not evaluated here, the economic burden of unnecessary carbapenem use warrants future pharmacoeconomic analysis.

## Conclusions

In this propensity score-matched study, pediatric patients with susceptible *E. coli* BSI who received third-generation cephalosporins had similar short-term outcomes as those who received inappropriate carbapenems. Starting with a narrower spectrum agent is not inferior to carbapenems. Therefore, continuing or starting carbapenems when susceptibility to cephalosporins is known provides no clinical advantage. Antimicrobial stewardship could prioritize initial narrow-spectrum therapy to reduce unnecessary carbapenem exposure and the long-term risk of resistance.

## Data Availability

The data presented in this study are available from the corresponding author upon reasonable request.
