# Supplementary material for: No improvement in clinical outcomes: a propensity score analysis of inappropriate carbapenem use in children with susceptible Escherichia coli bloodstream infections
Source: Front Cell Infect Microbiol. 2026 Jun 2;16:1829667. doi: 10.3389/fcimb.2026.1829667 (PMC13269023; doi:10.3389/fcimb.2026.1829667)
Supplement: Supplementary file 1 [file DataSheet1.docx]

**
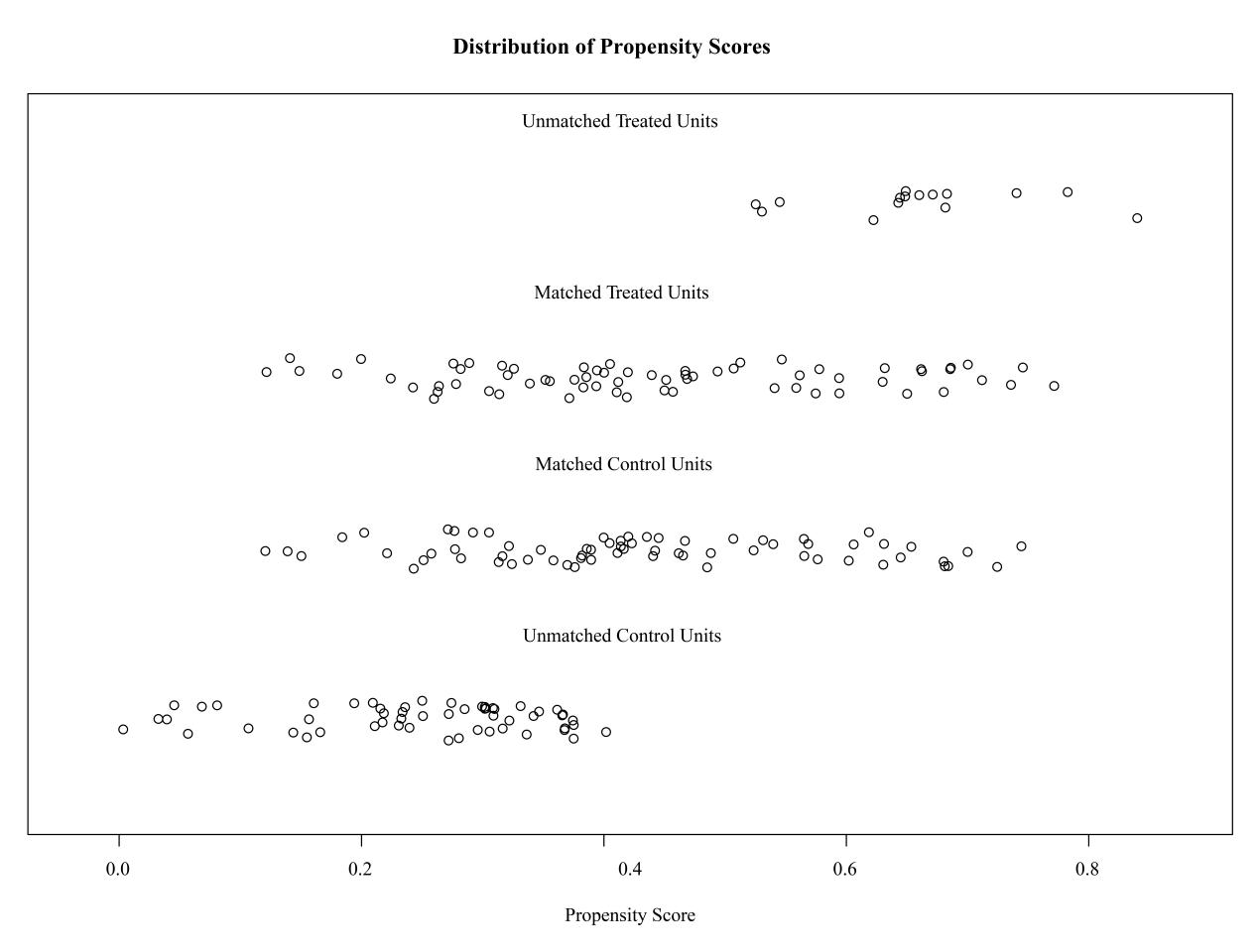
**Figure S1 Propensity score distribution before and after matching.****

**
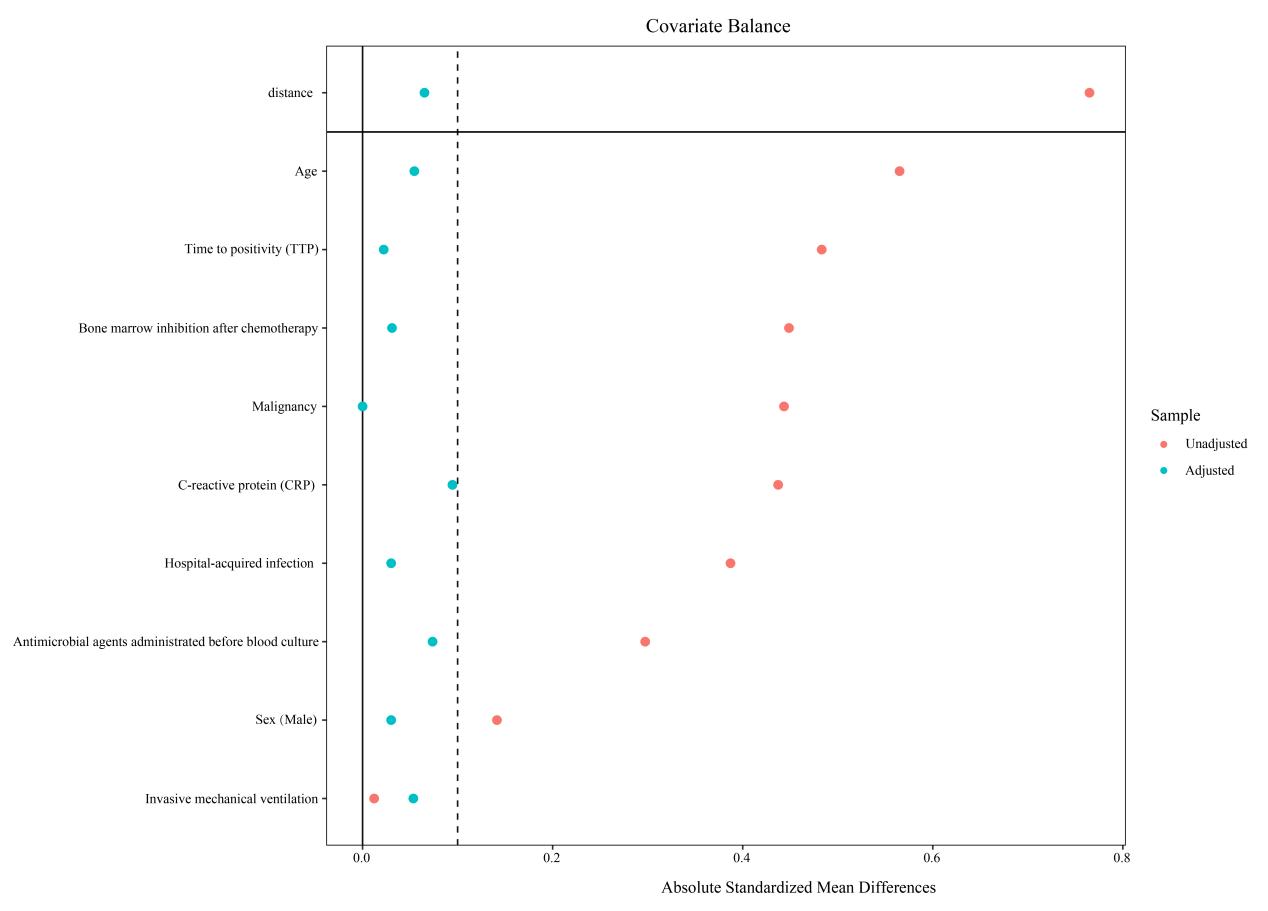
**

****Figure S2. Love plot of standardized mean differences before and after matching.****

****
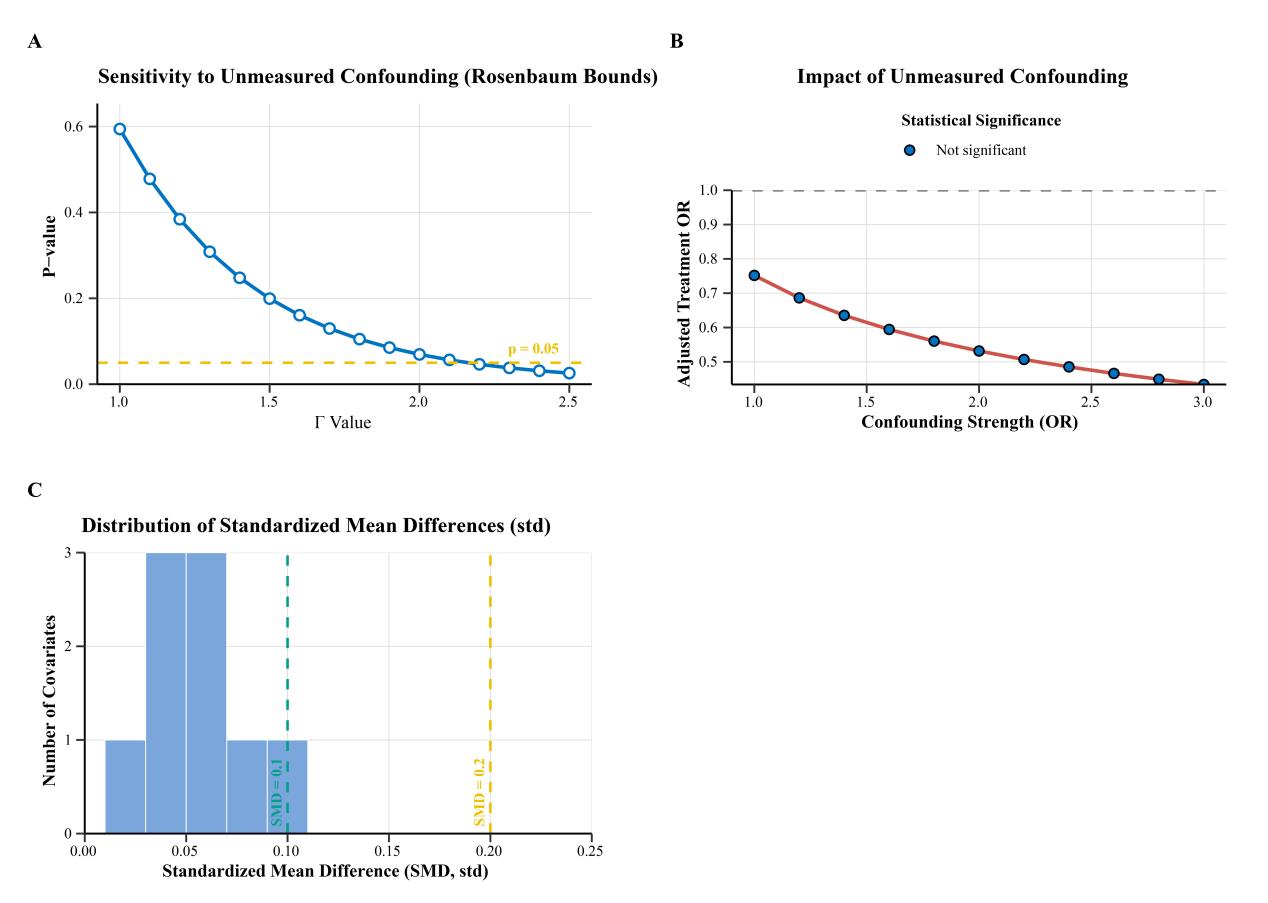
****

****Figure S3. Sensitivity analyses for unmeasured confounding.**(A) Rosenbaum bounds analysis. (B) Simulation of unmeasured confounders. (C) Distribution of post-matching standardized mean differences.**

****Table S1 Sensitivity analysis of clinical outcomes before and after excluding TTP in propensity score matching (PSM)****

| ****Outcome**** | ****With TTP**** | ****Without TTP**** |
| --- | --- | --- |
| ****Mortality**** | **OR = 0.75 (95%CI: 0.25-2.15), P = 0.595** | **OR = 1.00 (95%CI: 0.35-2.89), P = 1.000** |
| ****Sepsis**** | **OR = 0.92 (95%CI: 0.42-2.03), P = 0.841** | **OR = 1.00 (95%CI: 0.46-2.18), P = 1.000** |
| ****Septic shock**** | **OR = 0.82 (95%CI: 0.23-2.86), P = 0.753** | **OR = 1.00 (95%CI: 0.30-3.36), P = 1.000** |

****Table S2 Comparison of duration of hospitalization between groups before and after PSM****

| ****Matching Strategy**** | ****Group**** | ****n**** | ****Duration of hospitalization (IQR, days)**** | ****Test**** | ****p**** |
| --- | --- | --- | --- | --- | --- |
| **Before PSM (Full sample)** | **Carbapenem-Inappropriate** | **82** | **26.10 (15.30-35.70)** | **Wilcoxon rank-sum** | **0.067** |
|  | **Standard-Therapy** | **122** | **18.77 (12.54-36.15)** |  |  |
| **After PSM (with TTP)** | **Carbapenem-Inappropriate** | **67** | **23.92 (15.39-34.94)** | paired **Wilcoxon rank-sum** | **0.776** |
|  | **Standard-Therapy** | **67** | **26.96 (12.59-39.00)** |  |  |
| **After PSM (without TTP)** | **Carbapenem-Inappropriate** | **68** | **23.38 (14.47-34.79)** | paired **Wilcoxon rank-sum** | **0.553** |
|  | **Standard-Therapy** | **68** | **27.46 (13.55-38.98)** |  |  |
